# *De Novo* Design of Antimicrobial Peptides With a Special Charge Pattern and Their Application in Combating Plant Pathogens

**DOI:** 10.3389/fpls.2021.753217

**Published:** 2021-09-30

**Authors:** Eric H. -L. Chen, Cheng-Wei Weng, Yi-Min Li, Ming-Chin Wu, Chien-Chih Yang, Kung-Ta Lee, Rita P. -Y. Chen, Chiu-Ping Cheng

**Affiliations:** ^1^ Institute of Biological Chemistry, Academia Sinica, Taipei, Taiwan; ^2^ Institute of Plant Biology, National Taiwan University, Taipei, Taiwan; ^3^ Department of Biochemical Science and Technology, National Taiwan University, Taipei, Taiwan; ^4^ Institute of Biochemical Sciences, National Taiwan University, Taipei, Taiwan; ^5^ Department of Life Science, National Taiwan University, Taipei, Taiwan; ^6^ Global Agriculture Technology and Genomic Science Master Program, National Taiwan University, Taipei, Taiwan

**Keywords:** antimicrobial peptides, bacteria, fungi, membrane permeability, plant diseases, plant protection

## Abstract

Plant diseases are important issues in agriculture, and the development of effective and environment-friendly means of disease control is crucial and highly desired. Antimicrobial peptides (AMPs) are known as potential alternatives to chemical pesticides because of their potent broad-spectrum antimicrobial activity and because they have no risk, or have only a low risk, of developing chemical-resistant pathogens. In this study, we designed a series of amphipathic helical peptides with different spatial distributions of positive charges and found that the peptides that had a special sequence pattern “BBHBBHHBBH” (“B” for basic residue and “H” for hydrophobic residue) displayed excellent bactericidal and fungicidal activities in a wide range of economically important plant pathogens. The peptides with higher helical propensity had lower antimicrobial activity. When we modified the peptides with a long acyl chain at their N-terminus, their plant protection effect improved. Our application of the fatty acyl-modified peptides on the leaves of tomato and Arabidopsis plants lessened the infection caused by *Pectobacterium carotovorum* subsp. *carotovorum* and *Botrytis cinerea*. Our study provides important insights on the development of more potent novel AMPs for plant protection.

## Introduction

The main source of food of the global population is agriculture. However, plants that serve as food crops are constantly threatened by microbial infection. Diverse bacterial and fungal pathogens invade food crops, and each of the pathogens can have an extensive range of plant hosts, leading to enormous losses in crop yield (Fletcher et al., 2006). Global crop production is estimated to suffer a total yield loss of 20–40% due to plant pathogen infection, which poses a great threat to food security (Oerke, 2006). The application of chemical pesticides and the breeding of resistant crops are currently the most used means of disease control in plants. However, the use of chemical pesticides, including fungicides, bactericides, and nematicides, is environmentally harmful and may be toxic or even carcinogenic. Furthermore, pathogens can develop resistance to pesticides. On the other hand, breeding-resistant crops also have several setbacks, including the high cost of labor and the time needed to develop the resistant crops, the shortage of plant resistance genes, the easy overcoming of resistance genes by pathogens, and the limited defense spectrum (Louwaars, 2018). An effective and versatile means of pathogen control are still unavailable. Therefore, innovative, nontoxic, and nonpolluting antimicrobial means are urgently needed.

Antimicrobial peptides (AMPs) are small defense peptides naturally produced by a wide range of organisms. Thousands of AMPs have been identified from bacteria, fungi, animals, and plants (Zhao et al., 2013). They serve as the first line of defense in the innate immune system of these organisms against microbial intruders (Hancock and Sahl, 2006; Bolintineanu and Kaznessis, 2011; Schroeder et al., 2011; Ramos et al., 2013; Kumar et al., 2018). AMPs possess direct microbicidal, microbiostatic, or immunogenic effects (Andersson et al., 2016; Kumar et al., 2018). They usually consist of less than 50 amino acids and have diverse sequences (Haney and Vogel, 2009). Due to the differences in the eukaryotic and prokaryotic membrane construction, AMPs display selectivity for microbes, and thus, toxic side effects against cells of higher organisms are minimized. Therefore, AMPs are a promising alternative to conventional antibiotics and pesticides in combatting pathogens while ensuring human and plant health (Montesinos, 2007; Montesinos and Bardaji, 2008; Keymanesh et al., 2009; Meng et al., 2010; Jung and Kang, 2014; Mahlapuu et al., 2016; Lei et al., 2019).

Although AMPs have common features, such as of being cationic and amphipathic and of being rich in disulfide bonds, their activities and targets are difficult to predict from their sequences (Boman, 2003; Haney and Vogel, 2009; Akaddar et al., 2010; Liang et al., 2018). Most AMP designs are based on existing AMPs and are screened through trial and error (Chou et al., 2008; Rathinakumar and Wimley, 2010; Nguyen et al., 2011; Zeitler et al., 2013). It is known that not all the cationic and amphipathic peptides have desired antimicrobial activity. Moreover, many AMPs have antibacterial activity *in vitro* but not *in vivo* (Zeitler et al., 2013). Another problem with the agricultural application of AMPs is its high cost. To reduce the cost of peptide synthesis, the peptide length should be as short as possible. In addition, since orthogonal synthesis requires protected amino acids, cheaper protected amino acids are thus preferred. For example, both lysine (Lys) and arginine (Arg) are basic amino acids, but the price of Fmoc-Lys(Boc)-OH (Fmoc: the fluorenylmethoxycarbonyl protecting group) is much lower than that of Fmoc-Arg(Pbf)-OH.

For amphipathic AMPs, it has been proposed that AMPs have hydrophobic residues localized on one side of the molecule and cationic and polar residues localized on the opposite side (Nguyen et al., 2011). In this study, we hypothesized that the spatial arrangement of positively charged residues is important for the efficacy of AMPs. We designed *de novo* a series of AMPs with different charge patterns and various modifications and evaluated their antimicrobial efficacy against a range of important bacterial and fungal pathogens. Our results revealed the broad-spectrum efficacy of a group of our AMPs in suppressing the growth of pathogens *in vitro* and the development of plant diseases.

## Materials and Methods

### Peptide Synthesis

The peptides were synthesized on a PS3 peptide synthesizer (Protein Technologies, Inc., Arizona, United States) using the Fmoc-polyamide method (Lin et al., 2016; Chen et al., 2021). The C-terminus of the peptides was amidated using the Rink Amide AM resin (200–400 mesh, Novabiochem, Germany) as the solid support during the synthesis. The N-terminus of the peptides was protected using the corresponding acids instead of an amino acid derivative in the final synthetic step. Side-chain deprotection and peptide cleavage were performed by stirring the resin in a mixture of trifluoroacetic acid/water/ethanedithiol (95/2.5/2.5, v/v) at room temperature for 2h. After the cleavage, the resin was removed by filtration, and the peptides were precipitated by adding 10 volumes of ice-cold methyl t-butyl ether (MTBE) to the filtrate. The precipitate was collected by centrifugation at 3,000 × g for 15min at 4°C, washed twice with ice-cold MTBE, and vacuum-dried to get the crude peptides. Peptide purification was performed through reverse-phase high-performance liquid chromatography (RP-HPLC) using a C18 column (10mm×250mm, 10μm, SUPELCO, Sigma-Aldrich, Germany). The eluted peptide solution was collected, lyophilized, and stored in a−30°C freezer. The peptides were identified using matrix-assisted laser desorption ionization time-of-flight (MALDI-TOF) mass spectrometry (AutoFlex III Smartbeam, Bruker, United States; Supplementary Figure S1).

### Circular Dichroism Spectroscopy

The peptides were dissolved in water to make a stock solution and then diluted in the solution that contained different concentrations of trifluoroethanol (TFE) to the desired final TFE concentration. The circular dichroism (CD) spectra between 190 and 250nm were recorded on a J-815 CD spectrometer (JASCO, Japan). The bandwidth was 2nm, and the step resolution was 0.05nm. Each sample was scanned twice, and the average of the two measurements was smoothed using the Savitzky-Golay method to get the final CD spectrum. The CD spectra were deconvoluted using the CD Multivariate SSE software version 2.0.1 (JASCO, Japan).

For the CD spectra of the peptide pepD in lipids, two kinds of liposomes, DOPC and POPE/POPG (1:1, w/w), were prepared. DOPC (10mg/ml) and POPE/POPG (14mg/ml) were individually dissolved in chloroform/methanol (9/1, v/v) in a glass tube. The solvents were evaporated through nitrogen purging to form a thin lipid film on the tube surface. The tube was placed *in vacuo* overnight to completely evaporate the organic solvent. To rehydrate the lipid film, 400μl of deionized water was added, and the solution was mixed in an Intelli-mixer (rocker mode, 60rpm) for 1h. Then, the water/lipid mixture was frozen in liquid nitrogen and thawed at 45°C for 5min. After five freeze-thaw cycles, liposomes were prepared by extruding the mixture through a polycarbonate filter (with a 200-nm pore size) using an Avanti Mini-Extruder (Avanti Polar Lipids, United States). The purified pepD was dissolved in water (64μg/ml) and mixed with an equal volume of the liposome suspension for CD measurement.

### Microbes and Growth Conditions Used

The media and growth conditions for the test plant pathogens and the *Escherichia coli* strain DH5α are described in Supplementary Table S1. The phytopathogenic bacteria included *Xanthomonas euvesicatoria* (*Xev*) strain Xvt28, *Xanthomonas campestris* pv. *campestris* (*Xcc*) strain Xcc17, *Xanthomonas oryzae* pv. *oryzae* (*Xoo*) strain Xoo28, *Agrobacterium tumefaciens* (*Atu*) strain C58C1, *Pectobacterium carotovorum* subsp. *carotovorum* (*Pcc*), *Erwinia chrysanthemi* (*Ech*), *Pseudomonas syringae* pv. *tomato* (*Pst*), *Pseudomonas syringae* pv. *syringae* (*Pss*) strain B728a, and *Ralstonia solanacearum* (*Rs*) strains Pss4 and Pss190. Phytopathogenic fungi include as: *Colletotrichum gloeosporioides* (*Cgl*) strain FST02 and *Botrytis cinerea* (*Bc*).

### Determination of IC_50_ Against Bacteria

Single colonies grown on an optimal rich agar medium for each bacterium were picked and cultured in 3ml of the same rich liquid medium for 4~6h. Then, the bacterial culture was diluted in the same medium to produce a final concentration of OD_600_=0.08 and then further diluted 20-fold in the same medium. In each well of a 96-well polystyrene plate, 90μl of the diluted culture was mixed with 10μl of the peptide to produce peptide concentrations of 0, 2, 10, and 20μg/ml. The OD_600_ was measured after incubation at an optimal temperature for 24h. IC_50_ was determined as the lowest peptide concentration that inhibited 50% of the bacterial growth.

### Determination of the Minimal Inhibitory/Bactericidal Concentration Against Bacteria

Single bacterial colonies grown on an optimal rich agar medium were picked, and each colony grew in 3ml of the same rich liquid medium for 4–6h. For the experiments carried out in the rich medium, the bacterial broth culture was diluted in the same medium to produce a final concentration of OD_600_=0.08 and then further diluted 100-fold in the same medium. For the experiments carried out in the minimal medium, the bacterial cells were collected from the bacterial liquid culture through centrifugation, resuspended in an optimal minimal medium to produce a final concentration of OD_600_=0.08, and then further diluted 100-fold in the same minimal medium. Fifty microliters of the 2-fold serially diluted peptides was put on a 96-well polystyrene plate with 50μl of the bacterial culture. The final concentrations of the peptides were 0, 2, 4, 8, 16, and 32μg/ml. The plates were incubated at an optimal temperature without shaking. The minimal inhibitory concentration (MIC) was determined as the lowest concentration of the peptide at which no visible bacterial growth was observed after incubation for 48h in the rich medium or after incubation for 96h in the minimal medium. For the bacterium-peptide mixtures that did not have obvious bacterial growth, 10μl of the bacterium-peptide mixture was spotted on an optimal rich agar medium and incubated at an optimal temperature for 48h. The minimal bactericidal concentration (MBC) was determined as the lowest peptide concentration at which no colony was formed.

### Determination of the Minimal Inhibitory/Fungicidal Concentration Against Fungi

The fungal spore (10^6^ spores/ml) suspension in sterile water was diluted in the rich (PD) or minimal (CD) medium to produce a final concentration of 2×10^3^ spores/ml. Fifty microliters of the 2-fold serially diluted peptides was put in 96-well polystyrene plates with 50μl of the fungal spore suspension. The final concentrations of the peptides were 0, 2, 4, 8, 16, and 32μg/ml. The plates were incubated at an optimal temperature without shaking. The MIC was determined as the lowest concentration of peptides at which no visible fungal growth was observed after incubation for 72h in the rich medium or after incubation for 96h in the minimal medium. For the fungus-peptide mixtures that did not have obvious fungal growth, 10μl of the mixture was spotted on a PD plate and incubated at an optimal temperature for 24~72h. The minimal fungicidal concentration (MFC) was determined as the lowest peptide concentration at which no fungal growth was observed.

### Measurement of the Membrane Permeability of the Microbes

After the peptide treatments, the membrane permeability was analyzed using SYTOX Green staining. For the bacterium assays, the bacteria grown in the optimal rich liquid media for 16~20h were collected through centrifugation at 4,000×*g* and then resuspended and diluted in sterile water to produce a final concentration of OD_600_=0.4. The bacterial suspension (5μl) was mixed with 10μl of SYTOX Green (2μM) and 5μl of sterile water, Triton X-100, or the peptide (64μg/ml). For the fungal spore assays, the spores were collected from a fungal culture grown on PD plates for 7~10days and then put in sterile water. The spore suspension was filtrated with Miracloth, and the infiltrate was centrifuged to collect the spores. Then, the spores were suspended and diluted in sterile water to produce a final concentration of 10^6^ spores/ml (for the *Bc*) or 10^7^ spores/ml (for the *Cgl* FST02). The fungal spores (5μl) was mixed with 10μl of SYTOX Green (2μM) and 5μl of sterile water, Triton X-100, or the peptide (64μg/ml). For the fungal hyphae assays, 25μl of the fungal spores (10^5^ spores/ml) was mixed with 75μl of PD broth (pH=6.36~6.37), after which 5μl of the spore mixture was transferred to a glass slide and kept moist at room temperature in the dark for 24h to allow hyphal growth. Then, 10 microliters of SYTOX Green (2μM) and 5μl of sterile water, Triton X-100, or the peptide (64μg/ml) were added and kept moist. The SYTOX-Green-stained bacterial and fungal cells were observed using confocal microscopy (excitation light: 450–490nm, emission light: 500nm) after incubation at room temperature in the dark for 2h.

### Effects of the Peptide Applications on the Plant Disease Responses

Four-week-old tomato (*Solanum lycopersicum*) L390 and *Arabidopsis thaliana* Col0 plants grown at their optimal temperatures (25°C for the tomato and 21°C for the Arabidopsis) with a 12h/12h light/dark cycle were used for the pathogen inoculation assays.

For *Pcc*, the bacteria grown in the LB liquid medium at 28°C with shaking for 16~20h were collected by centrifugation at 4,000×g. The collected cells were resuspended in Buffer 1 (10mM MgSO_4_, 0.01% Silwet L-77) and then serially diluted in Buffer 2 (10mM MgSO_4_) to produce a final concentration of OD_600_=0.002. The *Pcc* suspension was mixed with the peptide (64μg/ml) at a 3:1 ratio to produce a final peptide concentration of 16μg/ml. Detached leaves of tomato plants and leaves of the intact Arabidopsis plants were wounded with a 10-μl micropipette tip and then droplet inoculated with 10μl of the *Pcc*-peptide mixture on the wounding sites. The detached tomato and Arabidopsis leaves were kept moist under their optimal growth conditions. Disease symptoms were photographed 16~28h post-inoculation, and the diameters of the lesions were measured.

For the *Bc* inoculation, the spores were collected from a fungal culture grown on a PD plate for 7~10days and then put in sterile water. The spore suspension was filtrated with Miracloth, and the infiltrate was centrifuged to collect the spores. Then, the spores were resuspended and diluted in sterile water to produce a final concentration of 10^6^ spores/ml, after which they were stored at −20°C. Prior to the inoculation, the *Bc* spore suspension was diluted to produce a final concentration of 10^4^ spores/ml for the tomato inoculation and 10^5^ spores/ml for the Arabidopsis inoculation. The diluted spore preparation was mixed with the peptide (64μg/ml) at a 3:1 ratio to produce a final peptide concentration of 16μg/ml. Detached tomato and Arabidopsis leaves were droplet inoculated with 10μl of the *Bc*-peptide mixture and kept moist under their optimal growth conditions. Disease symptoms were photographed 47~70h post-inoculation, after which the diameters of the lesions were measured.

## Results

### *De Novo* Design of AMPs and Their Antimicrobial Activity Against *Escherichia coli* and Plant Pathogens

**Figure 1:**
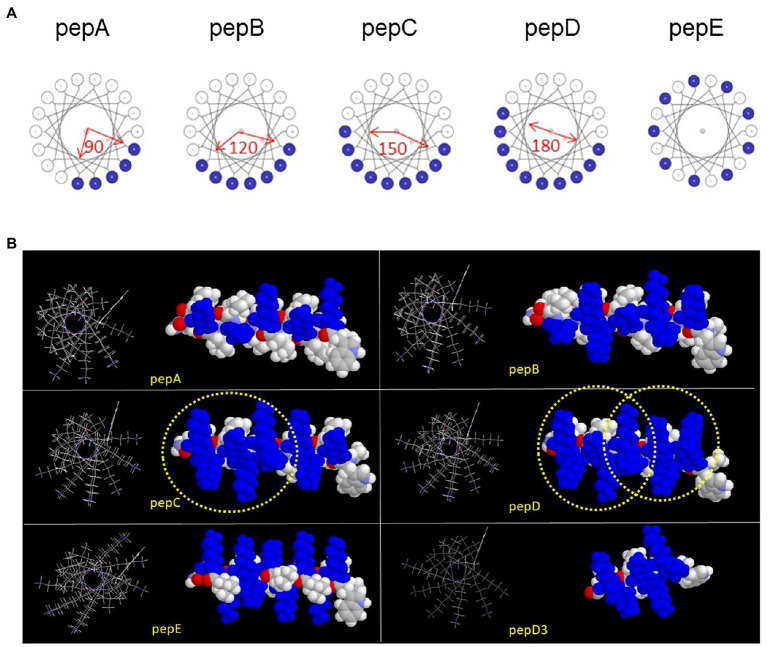
Designs and structural models of the *de novo* designed peptides. **(A)** Designs and structural models of the *de novo* designed peptides with different charge distribution patterns. **(B)** Structural models of the peptides in an α-helical conformation. The left image is the wireframe model of the helical wheel, and the right image is the space-filled model from the side of the helix. Lysine is colored blue. The “BBHBBHHBBH” sequence pattern is circled in pepC and pepD.

As shown in Table 1 and Figure 1A, five peptides, pepA, pepB, pepC, pepD, and pepE, were designed with different spatial distributions of their positively charged residues, assuming that the peptides had a α-helical conformation and the positively charged residues occupied a 90-degree, 120-degree, 150-degree, and 180-degree space in the α-helical conformation or were interspaced with the nonpositively charged residues. The modeled images of the charge distributions of the peptides are shown in Figure 1B.

**Table 1:** Peptides with different designs of the spatial distribution of positively charged residues.

Peptide	Positively charged residues		Sequence
	Percentage	Spatial distribution	
pepA	28%	90°	Ac-WKLLKLLLKLLKLLLKLL-NH_2_
pepB	39%	120°	Ac-WKLLKKLLKLLKKLLKLL-NH_2_
pepC	50%	150°	Ac-WKLLKKLLKKLKKLLKKL-NH_2_
pepD	56%	180°	Ac-WKKLKKLLKKLKKLLKKL-NH_2_
pepE	50%	Interspaced	Ac-WKLKLKLKLKLKLKLKLK-NH_2_

The crude peptides were further purified *via* HPLC. The antimicrobial activity of the HPLC-purified peptides was tested on the *E. coli* strain (*Eco*) DH5α and four phytopathogenic bacteria: *Xanthomonas euvesicatoria* (*Xev*) strain Xvt28, *Xanthomonas campestris* pv. *campestris* (*Xcc*) strain Xcc17, *Xanthomonas oryzae* pv. *oryzae* (*Xoo*) strain Xoo28, and *Agrobacterium tumefaciens* (*Atu*) strain C58C1. As shown in Table 2, the IC_50_ data showed that pepC, pepD, and pepE displayed a wide-range antimicrobial activity, whereas pepA and pepB had very low or no antimicrobial activity. These data show that these *de novo* designed AMPs have varied antimicrobial activities against *Eco* and the tested phytopathogenic bacteria.

**Table 2:** IC_50_ values of pepA, pepB, pepC, pepD, and pepE against *E. coli* and phytopathogenic bacteria.

Pathogen	Peptide (μg/ml)				
	pepA	pepB	pepC	pepD	pepE
*Eco* DH5α	–	–	10	10	10
*Xev* Xvt28	–	20	10	4	2
*Xcc* Xcc17	–	–	10	4	4
*Xoo* Xoo28	–	–	10	10	10
*Atu* C58C1	–	–	10	10	20

*“–” indicates that no antimicrobial activity was detected in rich media at the highest peptide concentration (20μg/ml) that was tested in this study*.

### Structural Characteristics of the Designed AMPs

**Figure 2:**
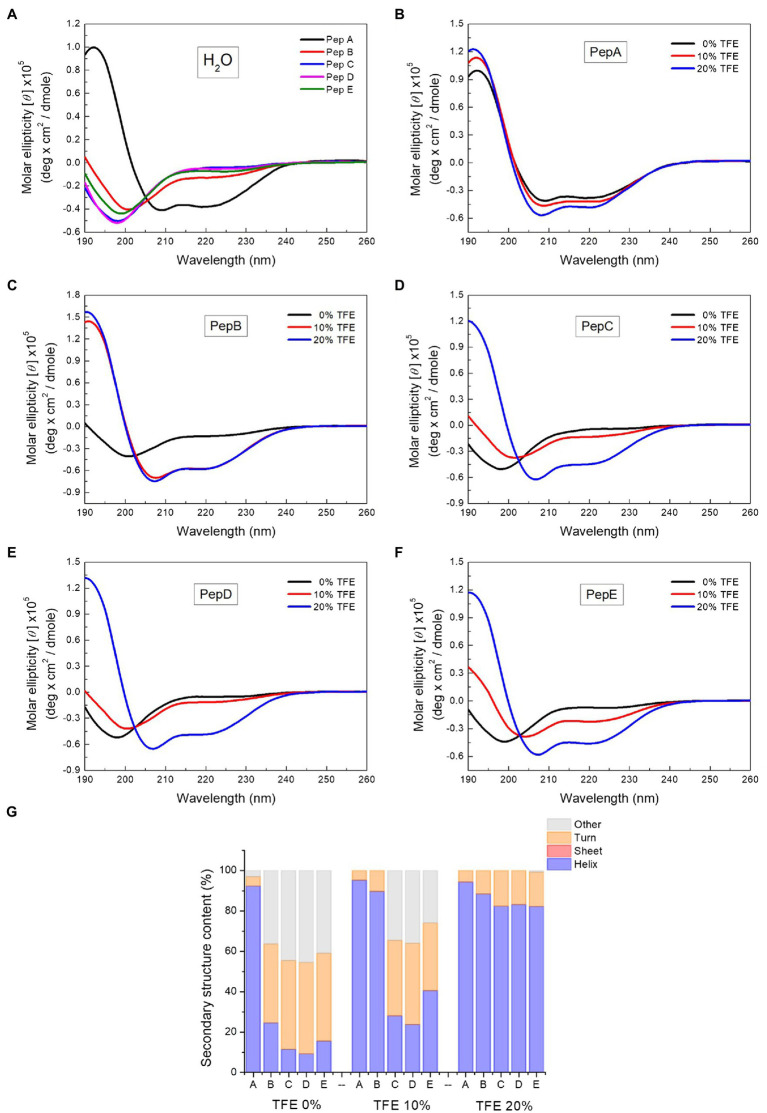
Circular dichroism (CD) spectra of the peptides and the spectrum deconvolution results. **(A)** CD spectra of pepA, pepB, pepC, pepD, and pepE in water. **(B–F)** CD spectra of the peptides in water, 10% trifluoroethanol (TFE), and 20% TFE. **(G)** Secondary structure content of the peptides in water, 10% TFE, and 20% TFE.

To investigate the relationship between the antimicrobial activities and the structural properties of the five peptides, their secondary structures were investigated *via* CD spectroscopy, which is commonly used to explore secondary structures of proteins and peptides. A negative peak at around 195nm in the CD spectrum indicates the existence of a random coil structure, whereas a positive peak at around 195nm, together with a negative peak at around 216nm, shows the presence of a β-sheet structure. The α-Helix structure is characterized by double negative peaks at 208 and 222nm and a positive peak at around 192nm. As shown in the CD spectra in Figure 2A, in water, pepA had an α-helix structure and pepB had a mixed α-helix structure and random coil, whereas pepC, pepD, and pepE had random coil structures in water. After structural deconvolution, pepA had over 90% α-helix content; pepB had only about 25% α-helix content; and the other three peptides had an α-helix content lower than 20% when they were dissolved in water (Figure 2G). The results showed that with an increase in the ratio of positively charged residues, the structural propensity to form an α-helix structure decreases.

To further explore the structural characteristics of the peptides, we next determined their CD spectra in TFE, a solvent that is commonly used to induce α-helix formation by promoting intramolecular hydrogen bond formation (Sonnichsen et al., 1992). The CD spectra of the peptides in water, 10% TFE, and 20% TFE (Figures 2B–F) showed that with the increase in the TFE concentration, a two-state structural transition from a random coil to an α-helix was observed for pepB, pepC, pepD, and pepE. An isodichroic point was found at 202nm (Figures 2C–F). 10% TFE was enough to induce 90% α-helix formation in pepB, and 20% TFE was needed to make the structures of pepC, pepD, and pepE α-helical. The results of the structural deconvolution of the five peptides in different TFE concentrations revealed that the α-helix structural propensity is pepA >pepB >pepE >pepC ≈ pepD. These data, together with the antimicrobial activities shown in Table 2, point out that the α-helix structural propensity of the peptides is inversely correlated with their antimicrobial activity.

**Figure 3:**
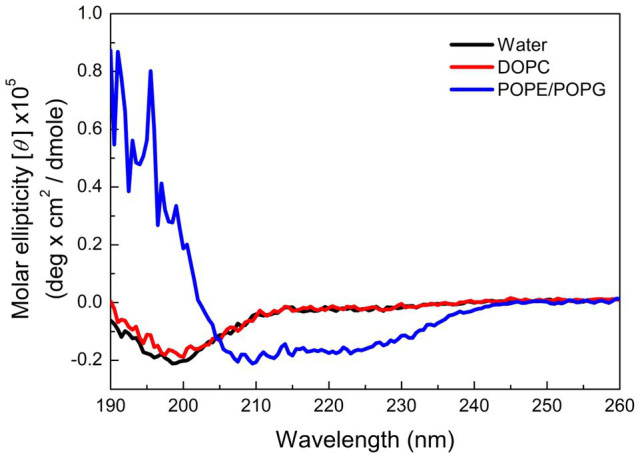
CD spectra of pepD in water (black) and in the presence of the DOPC liposomes (red) and the POPE/POPG liposomes (blue).

Many AMPs target microbial membranes for their antimicrobial actions (Nguyen et al., 2011). Since pepD had the best antimicrobial activity among the five peptides, we further examined its structural characteristics upon its interaction with lipids. 1,2-dioleoyl-sn-glycero-3-phosphocholine (DOPC) is commonly used to mimic the mammalian cell membrane. 1-Palmitoyl-2-oleoyl-sn-glycero-3-phosphoglycerol (POPG) has a negatively charged head group, and a mixture of 1-palmitoyl-2-oleoyl-sn-glycero-3-phosphoethanolamine (POPE) and POPG is commonly used to mimic the negatively charged membrane surface of bacteria. As shown in Figure 3, pepD retained its structure when it was mixed with water or DOPC, but it was induced to populate an α-helical structure when it was mixed with the POPE/POPG mixture. This suggests that pepD is lipid selective and interacts only with negatively charged lipids.

### Improvement of the Antimicrobial Activities of Shorter Peptide Varaiants of pepD

Cost is one of the most important factors of the application of AMPs. Since pepD was the most potent of the five peptides designed in this study, we further shortened its length and tested the antimicrobial activity of its short variants. Two peptides with four and seven residues that were shorter than pepD were synthesized and are referred to as pepD2 and pepD3, respectively (Table 3). The effect of the peptide length on the antimicrobial activity was investigated by determining the MIC, MBC, and MFC. The MIC and MBC data showed that the short variants of pepD maintained their antimicrobial activity and demonstrated even better activity than the original pepD (Table 4). For example, both pepD2 and pepD3 displayed MICs and MBCs against *Eco*, *Atu*, *Pcc, Pst,* and *Pss* that were lower than those of pepD. These results show that shortening pepD enhances its antibacterial activity. The peptide SP10-5 designed by Zeitler et al. (2013) was also included in the test for comparison (Table 4). The antibacterial activity of pepD2 and pepD3 had generally lower MICs and MBCs than that of SP10-5, which suggests that our peptides have better antibacterial activity.

**Table 3:** Variants of the pepD that was designed in this study.

Peptide	Carbon number of the N-end modification	Sequence
pepD	2	Ac-WKKLKKLLKKLKKLLKKL-NH_2_
Variants with reduced length		
pepD2	2	Ac-WKKLKKLLKKLKKL-NH_2_
pepD3	2	Ac-WKKLKKLLKKL-NH_2_
Variants with fatty acyl modifications		
pepD2M	14	Myristyl-WKKLKKLLKKLKKL-NH_2_
pepD3M	14	Myristyl-WKKLKKLLKKL-NH_2_
pepD2P	16	Palmitoyl-WKKLKKLLKKLKKL-NH_2_
pepD2S	18	Stearyl-WKKLKKLLKKLKKL-NH_2_
Variants with substituted hydrophobic residues		
pepI2	2	Ac-WKKIKKIIKKIKKI-NH_2_
pepV2	2	Ac-WKKVKKVVKKVKKV-NH_2_

**Table 4:** Minimal inhibitory concentrations (MICs) and minimal bactericidal concentrations (MBCs) of pepD, pepD2, and pepD3 against *E. coli* and phytopathogenic bacteria.

Pathogen	Peptide (μg/ml)							
	pepD		pepD2		pepD3		SP10-5	
	MIC	MBC	MIC	MBC	MIC	MBC	MIC	MBC
*Eco* DH5α	8	8	4	8	8	16	8	16
*Xev* Xvt28	4	16	4	4	4	4	8	8
*Xcc* Xcc17	2	8	1	1	2	2	2	2
*Xoo* Xoo28	4	4	4	8	4	4	8	8
*Atu* C58C1	4	–	2	2	2	2	2	4
*Pcc*	32	–	8	16	8	16	16	16
*Pst* DC3000	16	32	4	8	8	16	8	16
*Pss* B728a	32	–	8	16	8	16	8	32

*“–” indicates that no antimicrobial activity was detected in rich media at the highest peptide concentration (32μg/ml) that was tested in this study*.

However, at the highest peptide concentration (32μg/ml) that was tested in this study, pepD, pepD2, and pepD3 did not have detectable antimicrobial activity against a few bacterial and fungal plant pathogens, including the bacteria *Erwinia chrysanthemi* (*Ech*) and *Ralstonia solanacearum* (*Rs*), and the fungi *Botrytis cinerea* (*Bc*) and *Colletotrichum gloeosporioide*s (*Cgl*; data not shown). Since the nutrients available for the growth of plant pathogens under natural environments are poor, the antimicrobial activity of these AMPs was further tested in minimal media, which better mimic the real-world scenario of pathogen infection. Crude peptides were used for the assay to fit the real conditions for agriculture applications. As shown in Table 5, the inhibitory activity of pepD2 and pepD3 against *Ech* improved under a nutrient-limited condition, but these peptides still did not have detectable antimicrobial activity against *Rs*, *Bc*, and *Cgl* under the same conditions.

**Table 5:** MICs and MBCs/minimal fungicidal concentrations (MFCs) of pepD2, pepD3, and the corresponding myristylated peptide variants against phytopathogenic bacteria and fungi in minimal media.

Peptide (μg/ml)		Pathogen					
		*Pcc*	*Ech*	*Rs* Pss4	*Rs* Pss190	*Bc*	*Cgl* FST02
pepD2	MIC	8	16	–	–	–	–
	MBC/MFC	8	16	–	–	–	–
pepD2M	MIC	4	4	8	8	16	8
	MBC/MFC	4	8	8	8	16	16
pepD3	MIC	8	32	–	–	–	–
	MBC/MFC	16	-	–	–	–	–
pepD3M	MIC	4	4	8	16	4	8
	MBC/MFC	4	8	16	16	8	8

*“–” indicates that no antimicrobial activity was detected in minimal media at the highest concentration (32μg/ml) of crude peptides that was tested in this study*.

### Improvement of the Antimicrobial Activity of Peptide Variants With Fatty Acyl Modifications

The use of fatty acid modifications is favored for the application of AMPs to improve the interaction between the AMPs and microbial cell membranes. Due to the improved antimicrobial activity of pepD2 or pepD3, these peptides were subjected to fatty acid modifications (Table 3). Myristylated (C14), palmitoylated (C16), and stearylated (C18) variants of pepD2 are referred to as pepD2M, pepD2P, and pepD2S, respectively, and the myristylated variant of pepD3 is referred to as pepD3M. Surprisingly, although pepD2 and pepD3 did not show detectable antimicrobial activity against *Rs*, *Bc*, and *Cgl*, pepD2M and pepD3M displayed significantly improved antimicrobial activity against these pathogens under a nutrient-limited condition (Table 5). Furthermore, pepD2P had lower MIC and MFC values against *Bc* and *Cgl* than pepD2M in the minimal media, whereas pepD2S did not show detectable antimicrobial activity against these fungi (Table 6). These data indicate that the modification of the AMPs with fatty acids of a proper length augments the antimicrobial activity of the AMPs.

**Table 6:** MICs and MFCs of the fatty acyl-modified peptides against phytopathogenic fungi.

Pathogen	Peptide (μg/ml)					
	pepD2M		pepD2P		pepD2S	
	MIC	MFC	MIC	MFC	MIC	MFC
*Bc*	16	32	8	8	–	–
*Cgl* FST02	16	16	8	8	–	–

*“–” indicates that no antimicrobial activity was detected in minimal media at the highest peptide concentration (32μg/ml) that was tested in this study*.

### Improvement of the Antimicrobial Activity of Peptide Variants With Hydrophobic Residues That Have a Longer Aliphatic Chain

To find out if different hydrophobic residues affect antimicrobial activity, we synthesized and tested the antimicrobial activity of pepD2 variants that contained Leu, Ile, or Val as the hydrophobic residue (Table 3). pepI2 and pepV2 are the pepD2 variants with Ile and Val replacement, respectively. As shown in Table 7, pepI2 had a lower MIC and MBC against *Eco*, *Pcc*, and *Ech* than pepD2, whereas pepV2 had a lower or no detectable antimicrobial activity against these bacteria. These data indicate that the use of Ile, which has a longer aliphatic chain, as the hydrophobic residue enhances the antibacterial activity of AMPs.

**Table 7:** Antimicrobial activity of the peptides with different hydrophobic residues.

Pathogen	Peptide (μg/ml)					
	pepD2		pepI2		pepV2	
	MIC	MBC	MIC	MBC	MIC	MBC
*Eco* DH5α	8	8	4	4	32	32
*Pcc*	4	8	2	2	–	–
*Ech*	32	32	16	16	–	–

*“–” indicates that no antimicrobial activity was detected in rich media at the highest peptide concentration (32μg/ml) that was tested in this study*.

### Membranolytic Effect of AMPs on Pathogens

**Figure 4:**
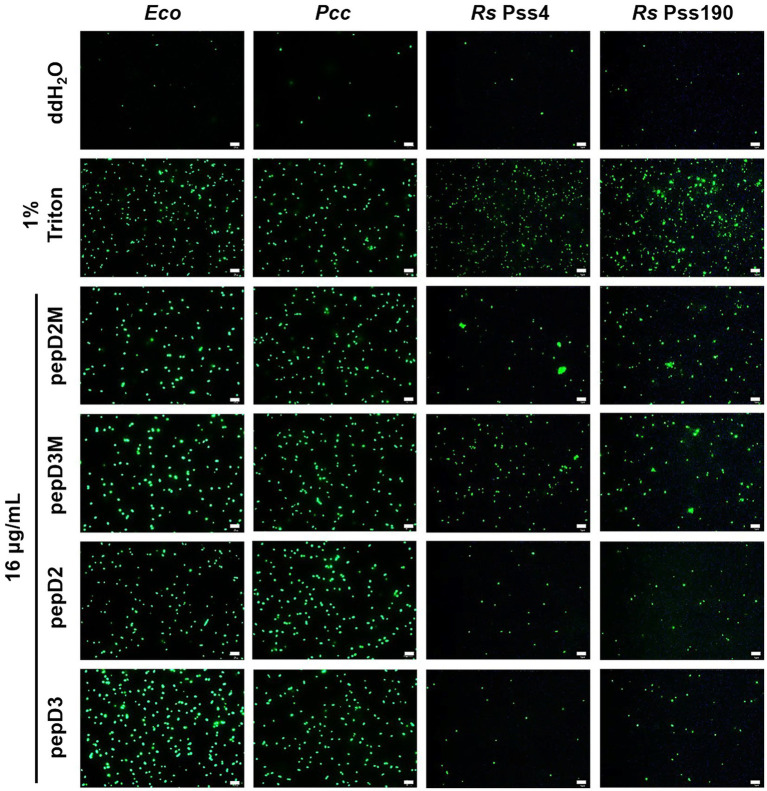
Effect of the peptides on the membrane permeability of *Escherichia coli* and phytopathogenic bacteria. The membrane permeability was analyzed after the peptide treatments using SYTOX Green staining. The bacteria that were cultured in optimal rich media for 16–20h were recovered and prepared as OD_600_=0.4 in sterile water. Five microliters of the bacterial suspension was mixed with 10μl of SYTOX Green (2μM) and 5μl of the peptide (64μg/ml). Crude peptides that were not purified *via* high-performance liquid chromatography (HPLC) were used. The SYTOX-Green-stained cells were observed using confocal microscopy after they were incubated for 2h in the dark. The data are from a single experiment that was repeated at least three times with similar results. *Eco*, *E. coli*; *Pcc*, *Pectobacterium carotovorum* subsp. *carotovorum*; and *Rs*, *Ralstonia solanacearum*. Bar=20μm.

**Figure 5:**
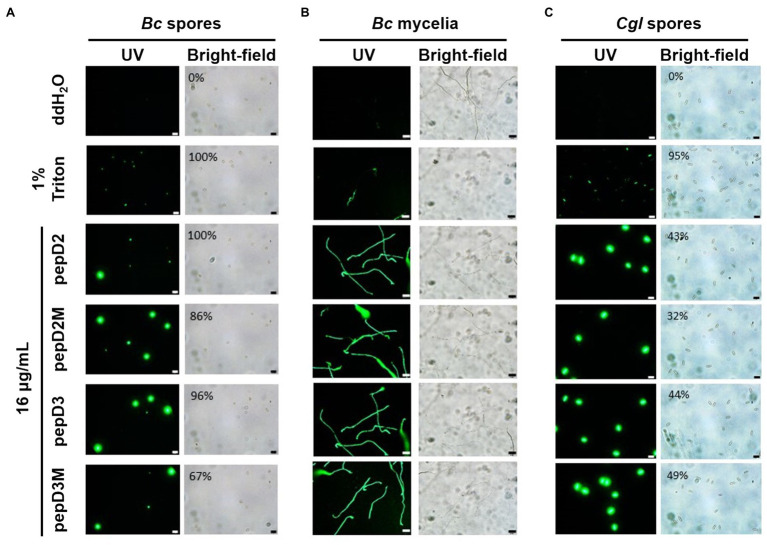
Effect of the peptides on the membrane permeability of phytopathogenic fungi. The membrane permeability was analyzed after the peptide treatments using SYTOX Green staining. The fungi *Botrytis cinerea* **(A,B)** and *Colletotricum gloeosporioides* **(C)** were cultured on optimal rich (PD) agar media for 7–10 days, and then, their spores were recovered and resuspended in sterile water. **(A,C)** Five microliters of the fungal spores (10^6^ spores/ml for **(A)** and 10^7^ spores/ml for **(C)** was mixed with 10μl of SYTOX Green (2μM) and 5μl of water, 4% Triton X-100, or pepD2M (64μg/ml). **(B)** Twenty-five microliters of the fungal spores (10^5^ spores/ml) was mixed with 25μl of PD broth (pH=6.36–6.37), after which 5μl of the spore mixture was transferred to a glass slide and kept moist at room temperature for 24h. Then, 10 microliters of SYTOX Green (2μM) and 5μl of water, 4% Triton X-100, or pepD2M (64μg/ml) were added to the mixture. Crude peptides that were not purified *via* HPLC were used. The SYTOX-Green-stained cells were observed *via* confocal microscopy after they were incubated for 2h in the dark **(A**–**C)**. In **(A,C)**, the ratios of the spores with SYTOX Green fluorescence are from a single experiment that was repeated at least three times with similar results. *Bc*, *Botrytis cinerea*; *Cgl*, *Colletotricum gloeosporioides*. Bar=20μm.

Many AMPs execute their antimicrobial actions by damaging the cell membrane (Hollmann et al., 2018). To investigate the mechanisms underlying the antimicrobial activity of our peptides, the membrane permeability of pathogens after AMP treatment was analyzed using SYTOX Green staining. This fluorescent dye cannot enter cells to stain the nucleic acids when the cell membrane is intact, whereas it can enter cells when the cell membrane is damaged and thus, the permeability is increased. pepD2, pepD3, pepD2M, and pepD3M were selected for the comparative analysis because of their noteworthy antimicrobial activity. As shown in Figure 4, the cells of the bacteria *Eco*, *Pcc,* and *Rs* were not stained with the dye in water, revealing the integrity of the cells. After treatment with Triton X-100, the bacterial cells were stained by the dye due to membrane damage. Treatment of the bacterial cells with pepD2, pepD3, pepD2M, and pepD2M all had a significant membranolytic effect on the test bacteria. In addition, the results of the cell-staining assays on the fungal spores and the mycelia showed that the treatments with these four crude peptides also damaged the *Bc* spores, the mycelia, and the *Cgl* spores (Figure 5). These data reveal that these AMPs have substantial membranolytic activity on bacterial and fungal cells.

### Reduction of Plant Diseases by AMPs

**Figure 6:**
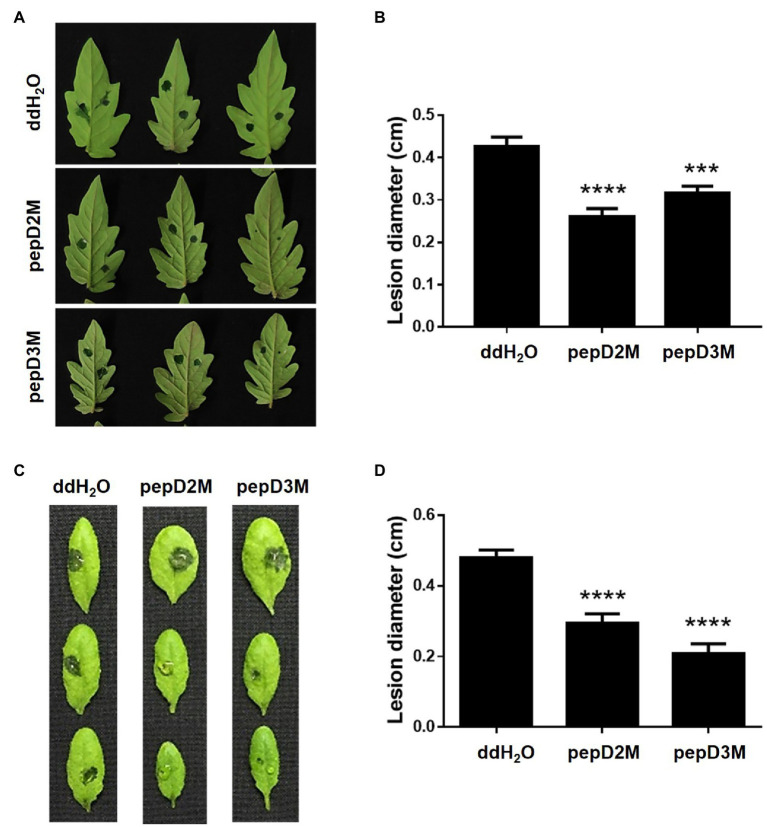
Effects of pepD2M and pepD3M on the plant responses after the infection with *Pectobacterium carotovorum* subsp. *carotovorum* (*Pcc*). The *Pcc* cell suspension (10^6^CFU/ml in 10mM MgSO_4_, 0.01% Silwet L-77) was mixed with the indicated peptide (64μg/ml) at a 3:1 ratio to produce a final peptide concentration of 16μg/ml. Crude peptides that were not purified *via* HPLC were used. Detached leaves of 4-week-old tomato L390 **(A,B)** plants and leaves of the intact Arabidopsis Col0 plants **(C,D)** were wounded with a 10μl micropipette tip and then droplet inoculated with 10μl of the *Pcc*-peptide mixture on the wounding sites. The disease symptoms were photographed 16–28h post-inoculation **(A,C)**, after which the diameters of the lesions were measured **(B,D)**. The data are from a single experiment that was repeated at least three times with similar results. **(B,D)** The values are the average±standard error (SE; n≥30). Pair-wise comparisons of the water- and peptide-treated samples were made using the Student’s *t*-test (^***^ *p*<0.001; ^****^ *p*<0.0001).

**Figure 7:**
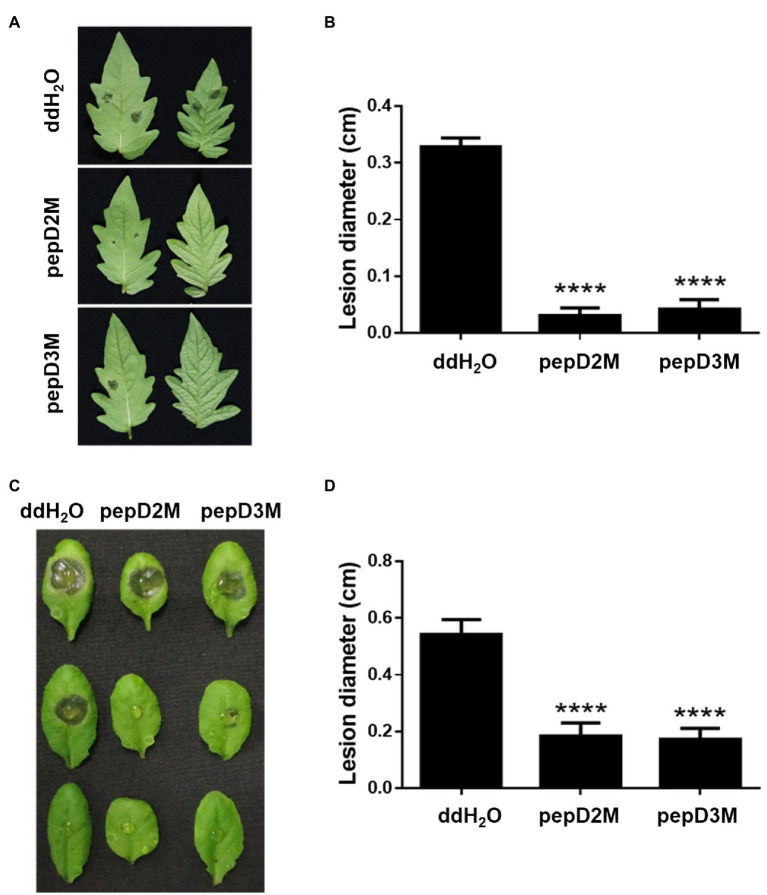
Effects of pepD2M and pepD3M on the plant responses after the infection with *Botrytis cinerea* (*Bc*). The *Bc* spore suspension (10^4^ spores/ml for the tomato inoculation and 10^5^ spores/ml for the Arabidopsis inoculation) was mixed with the indicated peptide (64μg/ml) at a 3:1 ratio to produce a final peptide concentration of 16μg/ml. Crude peptides that were not purified *via* HPLC were used. Detached leaves of 4-week-old tomato L390 plants **(A,B)** and leaves of the intact Arabidopsis Col0 plants **(C,D)** were wounded with a 10μl micropipette tip and then droplet inoculated with 10μl of the *Bc*-peptide mixture. The disease symptoms were photographed 47–70h post-inoculation **(A,C)**, after which the diameters of the lesions were measured **(B,D)**. The data are from a single experiment that was repeated at least three times with similar results. **(B,D)** The values are the average±SE (n≥30). Pair-wise comparisons of the water- and peptide-treated samples were made using the Student’s *t*-test (^****^ *p*<0.0001).

To further determine the *in planta* efficacy of AMPs in plant protection, the effects of selected AMPs on plant disease responses were analyzed. *Pcc* and *Bc* were chosen for the assays because they cause tremendous losses of many economically important crops worldwide, and they are among the top 10 bacterial and fungal pathogens, respectively, of crops (Dean et al., 2012; Mansfield et al., 2012). pepD2M and pepD3M were selected for the analyses because of their noteworthy antimicrobial activity. Crude peptides were used for the assays to mirror the real conditions for agriculture applications. The results showed that application of pepD2M and pepD3M significantly reduced the sizes of the disease lesions caused by *Pcc* on the Arabidopsis and tomato plants (Figure 6). The application of these two AMPs also decreased the development of the lesion caused by *Bc* (Figure 7). These results show that these fatty acyl-modified AMPs can inhibit the development of plant diseases caused by important bacterial and fungal plant pathogens.

## Discussion

### Importance of Using AMPs in Crop Disease Control

Crop losses due to various diseases caused by microbial pathogens have been huge worldwide, and global climate change is worsening the problem. The use of chemical pesticides and the breeding of resistant crops are the most used means of disease control. However, the use of chemical pesticides is damaging to the environment and human health and often leads to the emergence of resistant pathogens. Breeding of resistant crops also has bottlenecks (Louwaars, 2018). For devastating bacterial and fungal pathogens with a wide host range and ineffective means of chemical control, crop disease control is even more difficult, and thus, the resulting losses have been enormous. Therefore, alternatives to the management of crop diseases must be developed.

During their evolution, plants have become equipped with versatile defense mechanisms to cope with different stresses and sustain their life. The production of numerous plant AMPs is one of the most crucial components of the plant immune system. In plants, AMPs with microbicidal/microbiostatic activity are mostly cysteine-rich and can be classified into several families based on their structural characteristics (Campos et al., 2018; Iqbal et al., 2019). These AMPs are very stable due to their structural properties, and their role in defense against various microbial pathogens is well established in plants and animals (Campos et al., 2018; Iqbal et al., 2019). Because the action mode of a class of AMPs depends on the AMPs’ cationic and hydrophobic features, novel amphipathic AMPs can be rationally designed by directly modifying such features. Recently, synthetic *de novo* designed peptides have been shown to achieve the goals of breaking the signaling specificity barrier in different species and expanding the spectrum of antimicrobial activity (Zeitler et al., 2013; Datta et al., 2015; Hirakawa et al., 2017). Therefore, AMPs can be used as environment-friendly and effective agriculture products for plant protection.

### Structure-Activity Relationship of AMPs

In this study, we focused on designing amphipathic helical peptides against plant pathogens. To lower their cost, we used only three kinds of amino acids in the peptide design. Lys was used as the basic residue and Leu, as the hydrophobic residue, because they are cheaper than other amino acids with similar properties. One Trp was included for its high molar absorptivity at 280nm in peptide quantification. Assuming that the antimicrobial activity of AMPs is related to the helical propensity of the peptides, five lead peptides with five helical turns and different spatial patterns of positively charged residues were designed. Unexpectedly, the helical structural propensity was not proportional to the antimicrobial activity. On the contrary, the peptide that showed the strongest helical propensity, pepA, did not exhibit antimicrobial activity. The four other peptides could be induced to have a helical structure by adding TFE to them. The modeled images of the charge distributions of pepA, pepB, pepC, pepD, pepE, and pepD3 with a helical conformation are shown in Figure 1B. In pepC, pepD, pepD2, and pepD3, which showed better antimicrobial efficacy, we noticed a special sequence pattern, “BBHBBHHBBH” (“B” for basic and “H” for hydrophobic). This sequence pattern has three positively charged clips, and each clip contains two basic residues. These three positively charged clips form a special “triangle-shaped” cluster. PepC has only one positively charged triangle cluster, whereas pepD has two. The peptides with this sequence pattern can kill a broad range of important pathogens, including *Eco, Xev*, *Xcc*, *Xoo*, *Atu*, *Pcc*, *Ech*, *Pst*, *Rs, Bc*, and *Cgl*. When we decreased the peptide length of pepD, the shorter forms, pepD2 and pepD3, still had one positively charged triangle and maintained their excellent antimicrobial activity. We conclude that the spatial arrangement of basic residues is crucial for the bactericidal and fungicidal efficacy of AMPs. The CD spectra in Figure 2 showed that pepD can be transformed into an α-helical helix when it interacts with negatively charged liposomes. We surmise that the peptides with this sequence pattern can take away the negatively charged lipid molecules from the membrane using the positively charged clip cluster.

Could other hydrophobic amino acid residues replace Leu? Leu is the most frequently shown residue in the SWISS-PROT protein database (Garrett and Grisham, 2010). The side chains of Ile and Val are branching at the β-carbon, whereas Leu’s side chain shows branches at the γ-carbon. The hydrophobicity of Leu, Ile, and Val is Ile>Leu>Val (Mozhaev et al., 1988). This order is the same as the order of magnitude of the antimicrobial efficacy of these three peptides: pepI2>pepD2>pepV2 (Table 7). It suggests that hydrophobicity is vital for the observed antimicrobial effect.

Zeitler et al. have effectively designed a series of AMPs against various plant pathogens, such as *Xanthomonas vesicatoria, Pseudomonas syringae* pv. *tomato,* and *Pseudomonas syringae* pv. *syringae* (Zeitler et al., 2013). They designed four groups of amphipathic peptides. Group I has a dominant charged cluster; Group II has a dominant hydrophobic cluster; Group III has charged and hydrophobic regions with equal sizes; and Group IV has a hydrophobic cluster in the middle and a charged region at both ends. Among these groups, SP10-5 in Group III, which contains 42% positively charged residues, was the most effective. Our results further showed that SP10-5 displayed activities against additional bacteria, including *Eco*, *Xcc*, *Xoo*, *Atu*, and *Pcc* (Table 4). However, our pepD2 and pepD3 showed differentially better antibacterial activity than that of SP10-5.

### Significant Effect of Fatty Acyl Modifications on Antimicrobial Activity Against *Pcc*, *Ech*, *Rs*, *Bc*, and *Cgl*

Peptide stability is an important factor of the bioavailability of AMPs. To avoid AMP degradation by peptidases secreted by pathogens, in our original design, all the peptides were acetylated at the N-terminus and amidated at the C-terminus. To increase the interaction of the peptides and the lipids, myristoylation, palmitoylation, and stearylation were used to replace acetylation in pepD2 and pepD3. Table 5 shows that the myristylated AMPs kill bacteria and fungi more efficiently than the acetylated AMPs, suggesting that the length of the fatty acyl chain can affect antimicrobial activity. As shown in Table 6, the palmitoylated peptide pepD2P is even more effective than the myristylated peptide pepD2M. In contrast, the stearylated peptide pepD2S did not show antimicrobial activity in the peptide concentrations that we tested. We conclude that C14/C16 fatty acyl modification can be used to design AMPs for agricultural applications.

### Application of AMPs Against Pathogen Infection *in Planta*

Although a lot of AMPs have been studied, their antimicrobial activity was mostly tested at the *in vitro* level. Many AMPs display antimicrobial activity *in vitro* but fail to function on plants (Zeitler et al., 2013). For instance, the release of certain plant substances upon pest infection is seen as interfering with the activity or causing AMP degradation (Zeitler et al., 2013). In this study, we further tested the *in planta* efficacy of the two most potent AMPs, pepD2M and pepD3M, in reducing diseases caused by *Pcc* and *Bc*, both of which have an unusually wide host range (Dean et al., 2012; Mansfield et al., 2012). Chemical strategies, which were once used for the control of these two devastating pathogens, are no longer suggested due to two serious problems: the great influence on environmental and human health, and the high frequency of the development of multiple fungicide/bactericide resistance (Droby et al., 2009; Jess et al., 2014).

*Pcc* is a Gram-negative bacterium that causes the soft rot disease on hundreds of plant species (Mansfield et al., 2012). *Bc* is a necrotrophic fungal pathogen that causes the gray mold disease on hundreds of plant species and is ranked as the second most important phytopathogenic fungus (AbuQamar et al., 2017). Currently, no commercial peptides are available for the control of the diseases. In this study, we showed that pepD2M and pepD3M not only have significant *in vitro* anti-*Pcc* and anti-*Bc* activity (Table 5; Figures 4, 5), but also could reduce the development of the soft rot and gray mold diseases on tomato and Arabidopsis plants (Figures 6, 7; Supplementary Table S2), which demonstrate the efficacy of these AMPs in controlling the devastating pathogens. Furthermore, the results of parallel-line assays showed that pepD2 and pepD3 did not detectably inhibit the diseases caused by *Pcc* and *Bc* on tomato and Arabidopsis plants under the same experimental conditions (Supplementary Table S2). We thus conclude that the fatty acyl modification of AMPs improves both their *in vitro* antimicrobial activity and their efficacy in plant protection. The long fatty acyl chain may be able to enhance the retention of the peptides on the plants.

## Conclusion

In the use of AMPs as plant protectants for agricultural applications, cost and stability are the major limiting factors. The principles of AMP design include (1) choosing the cheapest aliphatic amino acid and basic amino acids; (2) using fatty acids for end modification to improve the peptide stability; and (3) looking for the balance between the peptide length and the antimicrobial efficacy. In this study, we showed that a group of peptides as short as 11-residues long with optimized modifications have a broad-spectrum efficacy in killing pathogens in media and suppressing the development of plant diseases. The safety evaluation of these AMPs has been described in our recent publication (Chen et al., 2021). These results are projected to benefit the development of novel peptide-based means of plant protection.

## Data Availability Statement

The original contributions presented in the study are included in the article/Supplementary Material, and further inquiries can be directed to the corresponding authors.

## Author Contributions

EC, C-WW, Y-ML, and M-CW conducted the experiments and analyzed the data. C-CY and K-TL discussed the results. RC and C-PC designed the experiments, analyzed the data, and wrote the manuscript. All authors contributed to the article and approved the submitted version.

## Funding

This study was supported by grants from the Ministry of Science and Technology (MOST) of Taiwan (MOST 106-3114-B-002-007, MOST107-2321-B-002-047, MOST 108-2321-B-002-052, and MOST 109-2313-B-002 -044 -MY3) and from National Taiwan University (NTU-CC-110L893606).

## Conflict of Interest

A provisional patent application (63117530) has been filed.

## Publisher’s Note

All claims expressed in this article are solely those of the authors and do not necessarily represent those of their affiliated organizations, or those of the publisher, the editors and the reviewers. Any product that may be evaluated in this article, or claim that may be made by its manufacturer, is not guaranteed or endorsed by the publisher.
