# Supplementary material for: De Novo Design of Antimicrobial Peptides With a Special Charge Pattern and Their Application in Combating Plant Pathogens
Source: Front Plant Sci. 2021 Sep 30;12:753217. doi: 10.3389/fpls.2021.753217 (PMC8514817; doi:10.3389/fpls.2021.753217)
Supplement: Supplementary file 1 [file Data_Sheet_1.pdf]

Supplementary Material

1 Supplementary Tables

SUPPLEMENTARY TABLE S1 | The pathogens and their growth conditions used in this study.

**Pathogen**	**Rich/minimal medium**	**Growth temperature**
Eco (*Escherichia coli*)	LB[3]/M9[4]	37°C
Xev (*Xanthomonas euvesicatoria*)	523[1]/-[1]	28°C
Xcc (*Xanthomonas campestris* pv. *campestris*)	523/-	28°C
Xoo (*Xanthomonas oryzae* pv. *oryzae*)	523/-	28°C
Atu *(Agrobacterium tumefaciens)*	YEP[2]/-	28°C
Pcc (*Pectobacterium carotovorum* subsp. *carotovorum*)	LB/M9	28°C
Ech (*Erwinia chrysanthemi*)	LB/M9	28°C
Pst (*Pseudomonas syringae* pv. *tomato*)	KB[5]/-	28°C
Pss (*Pseudomonas syringae* pv. *syringae*)	KB	28°C
Rs (*Ralstonia solanacearum*)	523/M9	28°C
Cgl (*Colletotrichum gloeosporioides*)	PD[6]/CD[7]	25–28°C
Bc (*Botrytis cinerea*)	PD/CD	25~28°C

[1]523: Bacterial rich medium 523. "-" indicates no minimal medium was used for the indicated pathogen in this study.
[2]YEP: Yeast extract peptone medium
[3]LB: Luria broth medium
[4]M9: M9 minimum medium
[5]KB: King's B medium
[6]PD: Potato dextrose medium
[7]CD: Modified Czapek-Dox (CD) minimal medium

SUPPLEMENTARY TABLE S2 | Effects of selected peptides on the plant disease responses after the infection with *Pectobacterium carotovorum* subsp. *carotovorum* (*Pcc*) and *Botrytis cinerea* (*Bc*).

**Peptide**	**Plant response to infection with:**			
	Pcc		***Bc***	
	**Tomato**	**Arabidopsis**	**Tomato**	**Arabidopsis**
pepD2	ns(3)	T*(1), ns(2), S*(1)	S*(1) S***(1)	ns(2)
pepD3	ns(5), S*(1)	T*(1), ns(4), S*(1)	ns(1), S***(1)	T**(1), ns(2)
pepD2M	T*(3), T**(1), T****(5)	T*(2), T***(4)	T*(1), T**(2), T***(1), T****(7)	T*(2), T***(4)
pepD3M	T**(2), T***(2), T****(2)	T**(1), T***(4)	T*(1), T**(1), T***(2), T****(8)	T*(1), T**(1), T***(4)

The *Pcc* cell suspension (10^6 CFU/mL in 10mM $MgSO_4$, 0.01% Silwet L-77) was mixed with the indicated peptide (64 µg/mL) at a 3:1 ratio to produce a final peptide concentration of 16 µg/mL. The *Bc* spore suspension (10^4 spores/mL for the tomato inoculation and 10^5 spores/mL for the Arabidopsis inoculation) was mixed with the indicated peptide (64 µg/mL) at a 3:1 ratio to produce a final peptide concentration of 16 µg/mL. Crude peptides that were not purified via high-performance liquid chromatography (HPLC) were used. Detached leaves of 4-week-old tomato L390 plants and leaves of the intact Arabidopsis Col0 plants were wounded with a 10µL micropipette tip and then droplet-inoculated with 10 µL of a pathogen-peptide mixture on the wounding sites. The diameters of the lesions were measured 16–28 h post-inoculation for *Pcc* and 47–70 h post-inoculation for *Bc*. Pair-wise comparisons of the water- and the peptide-treated samples were made using the Student's *t*-test ("ns", no significant difference; *$P < 0.05$, **$P < 0.01$, ***$P < 0.001$, ****$P < 0.0001$). "T" indicates that the peptide-treated plants were more tolerant to the pathogens than were the water-treated plants. "S" indicates that the peptide-treated plants were more susceptible to the pathogens than were the water-treated plants. The numbers in parentheses are the numbers of batches that showed the indicated result.

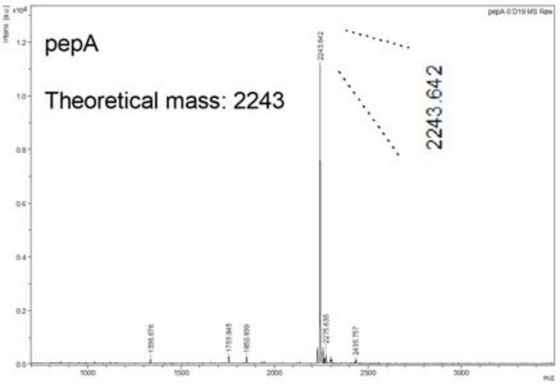
pepA
Theoretical mass: 2243
2243.642

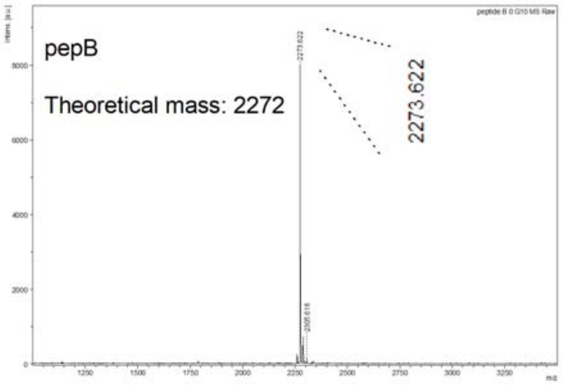
pepB
Theoretical mass: 2272
2273.622

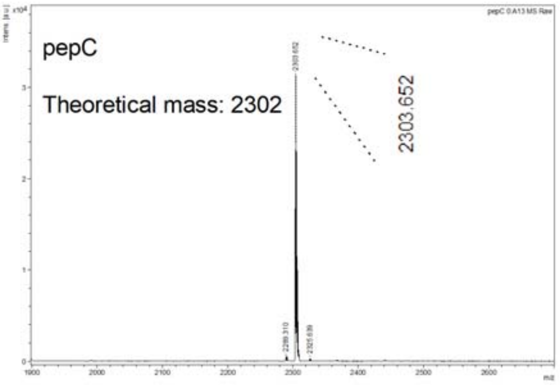
pepC
Theoretical mass: 2302
2303.652

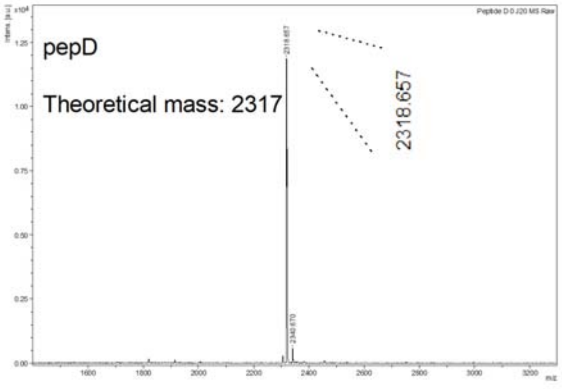
pepD
Theoretical mass: 2317
2318.657

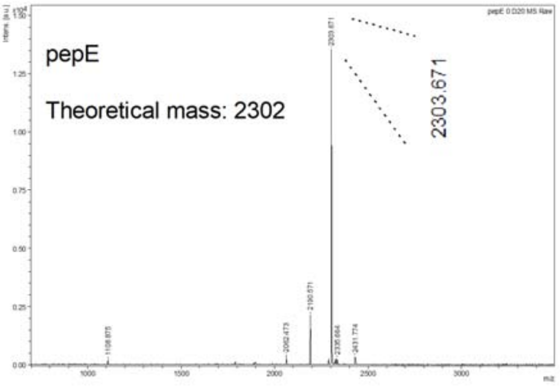
pepE
Theoretical mass: 2302
2303.671

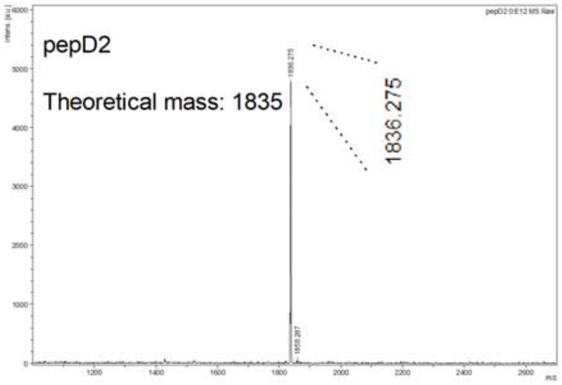
pepD2
Theoretical mass: 1835
1836.275

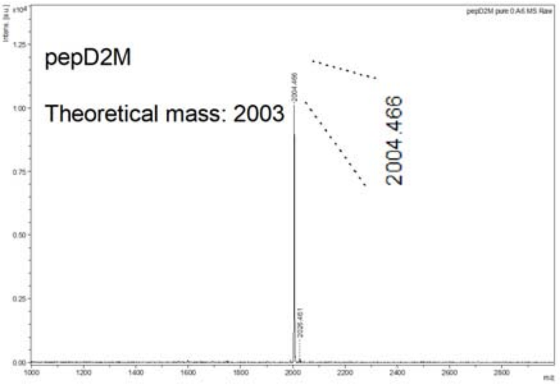
pepD2M
Theoretical mass: 2003
2004.466

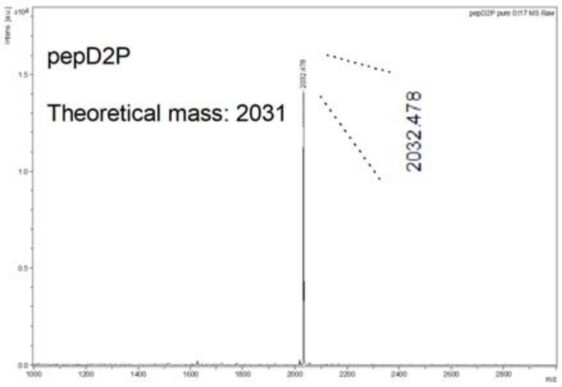
pepD2P
Theoretical mass: 2031
2032.478

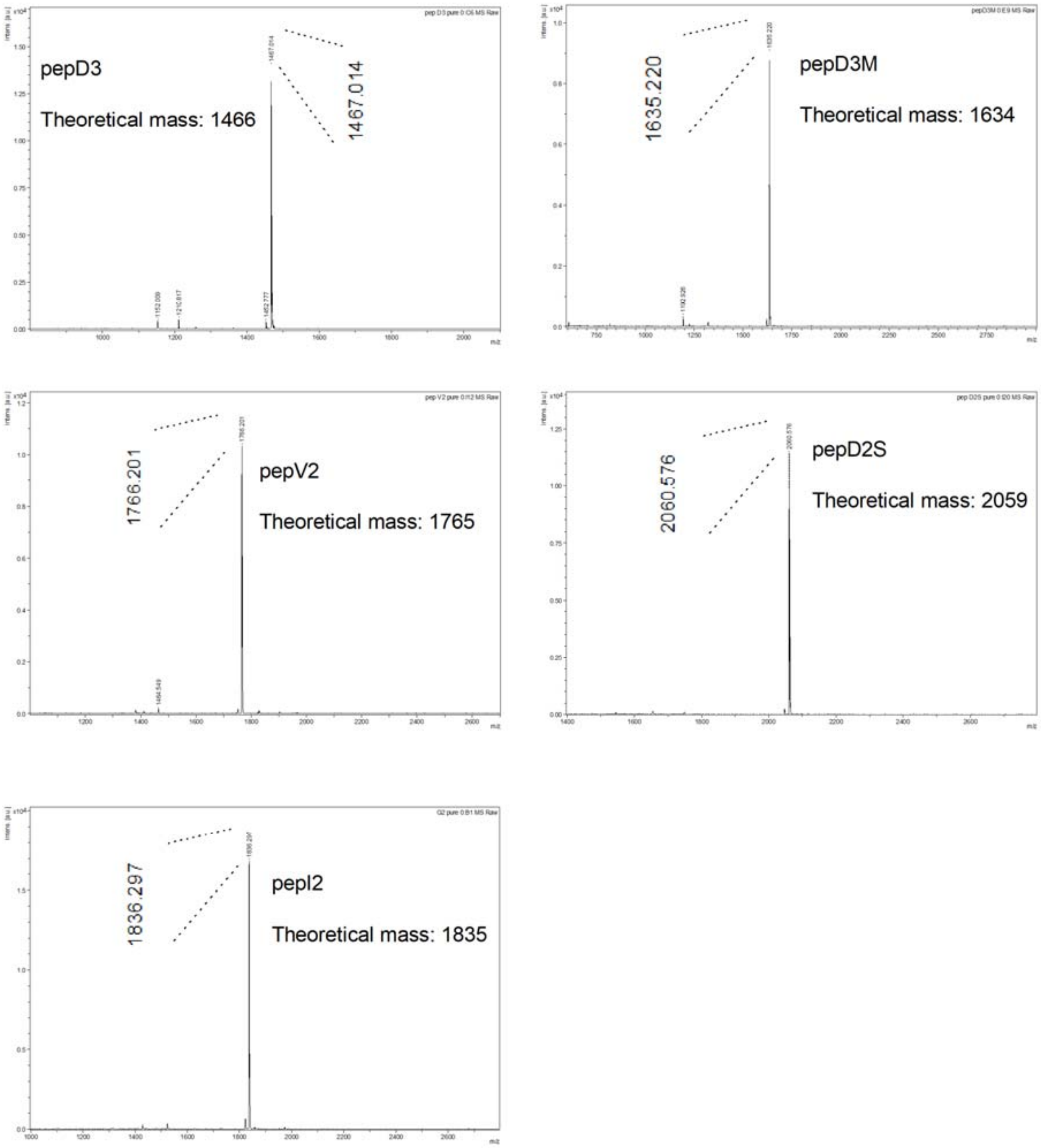

SUPPLEMENTARY FIGURE S1. Mass spectra of the peptides used in this study.
